# Supplementary material for: Global Variation in Escherichia coli mcr-1 Genes and Plasmids from Animal and Human Genomes Following Colistin Usage Restrictions in Livestock
Source: Antibiotics (Basel). 2024 Aug 12;13(8):759. doi: 10.3390/antibiotics13080759 (PMC11350921; doi:10.3390/antibiotics13080759)
Supplement: Supplementary file 1 [file antibiotics-13-00759-s001.zip › Supplementary_Figures.pdf]

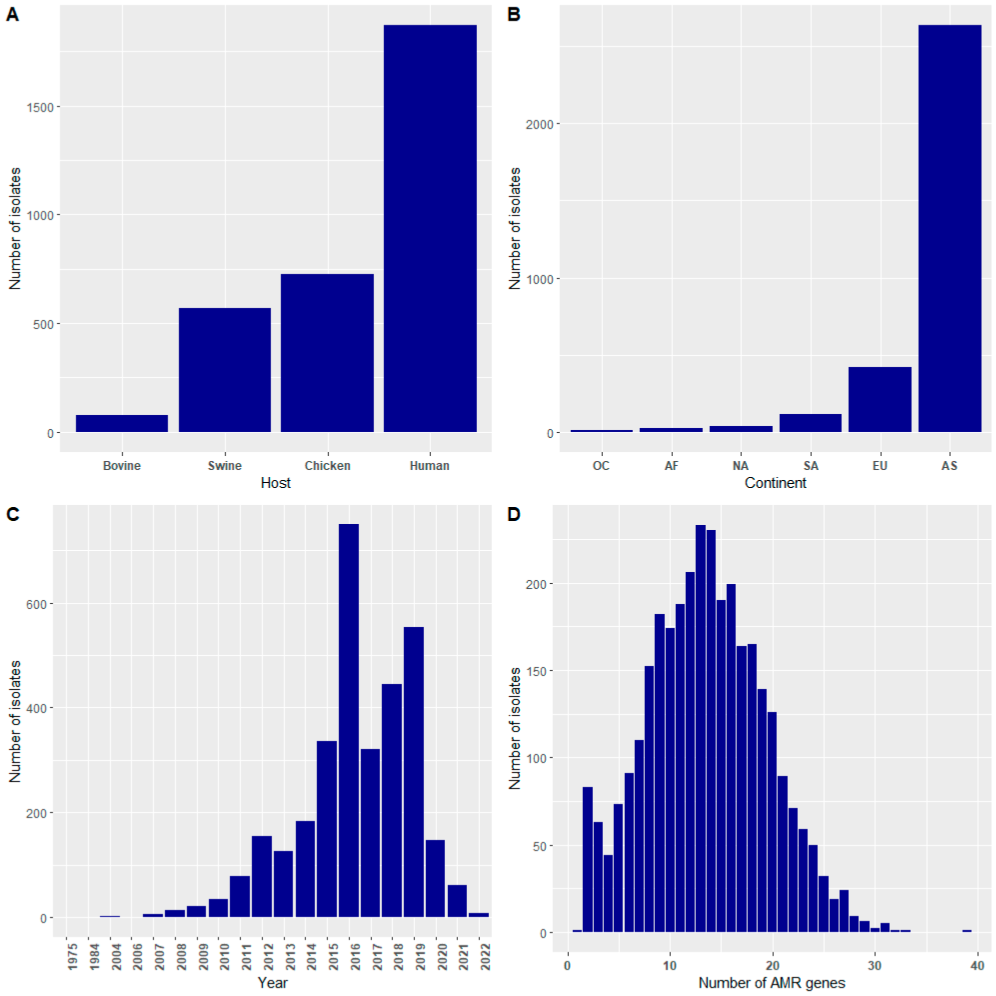

Supplementary Figure S1: Description of the general characteristics of the dataset. A: Count of isolates for each host. B: Distribution of the isolates in each continent. OC: Oceania. AF: Africa. NA: North America. SA: South America. EU: Europe. AS: Asia. C: Count of isolates for each year. D: Histogram of carrying of AMR genes.

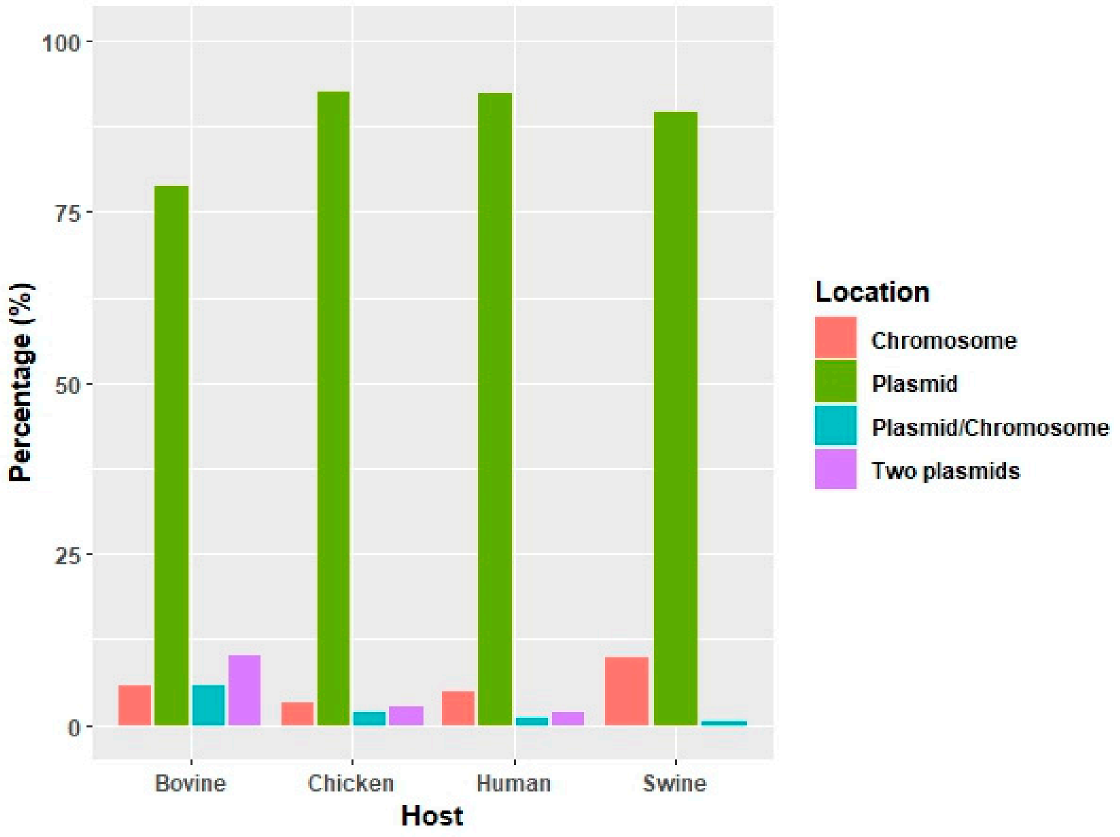

Supplementary Figure S2: Comparation of the genome location of *mcr*-1 gene between the different species. Localization is shown with different colours: red—chromosome, green—plasmid, blue--into a plasmid and also in the chromosome, purple--Into two plasmids in the same genome.

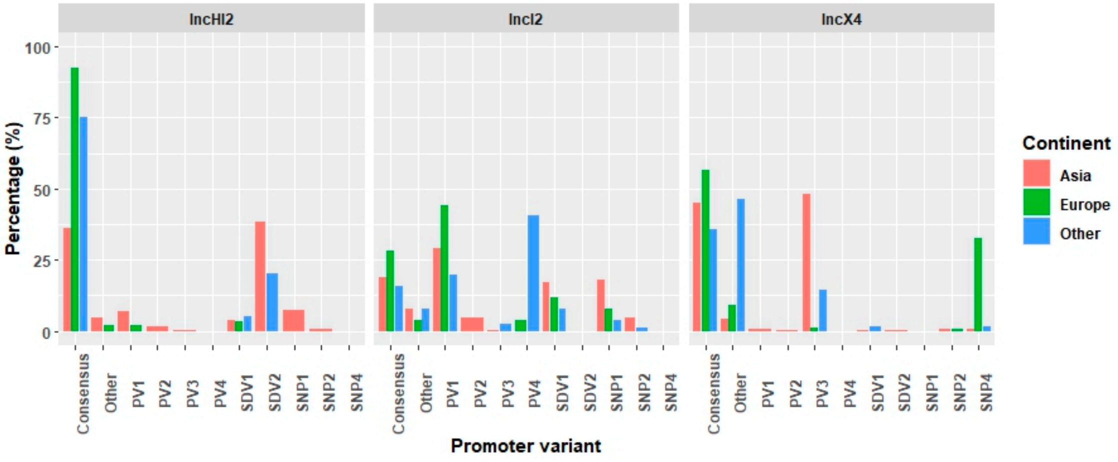

Supplementary Figure S3: Bar plots comparing the frequency of each promotor for the three most common plasmids in each continent. Each colour represents a distinct continent: red—Asia, green—Europe, blue—other continents.

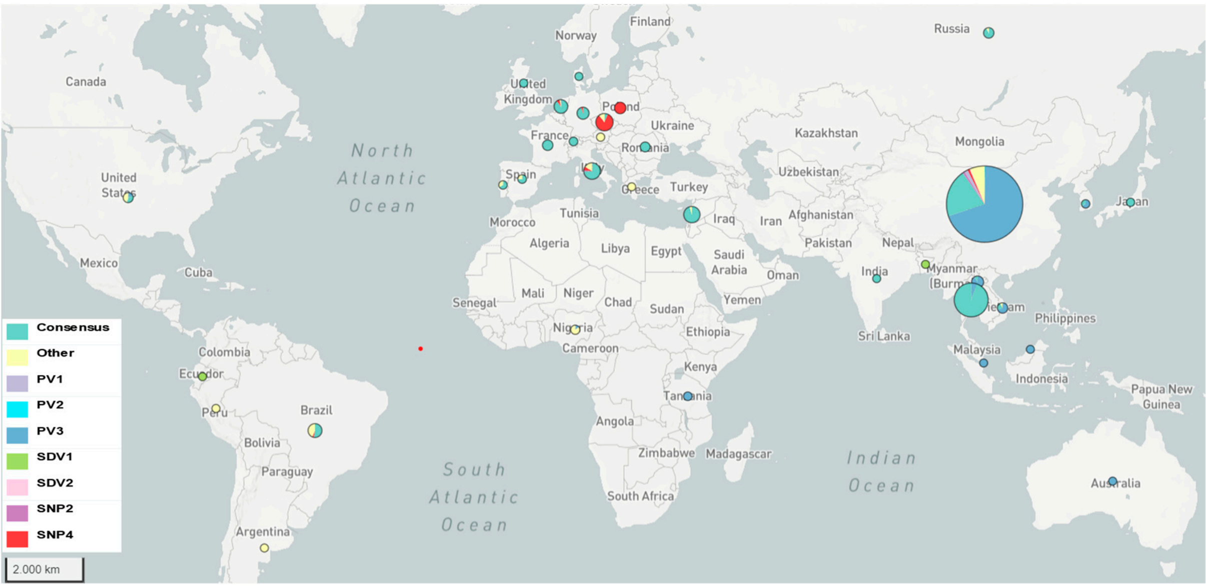

Supplementary Figure S4: Global map showing the frequency of promotor variant in each country for IncX4 plasmid. The pie chart size increases according to the number of genomes present in the specific country.

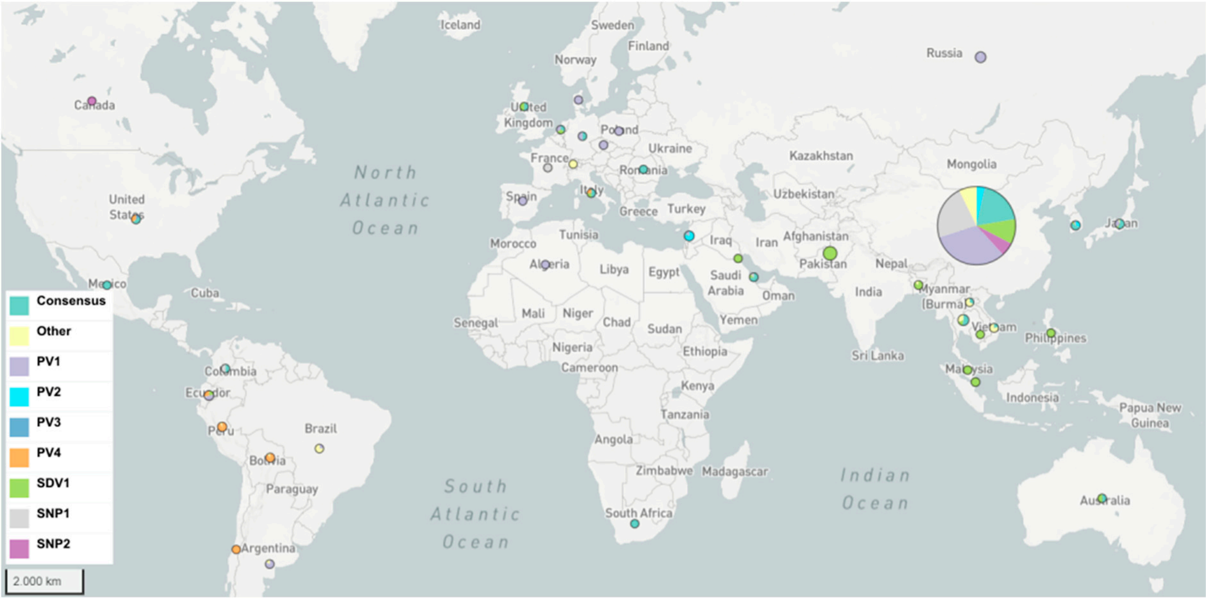

Supplementary Figure S5: Global map showing the frequency of promotor variant in each country for IncI2 plasmid. The pie chart size increases according to the number of genomes present in the specific country.

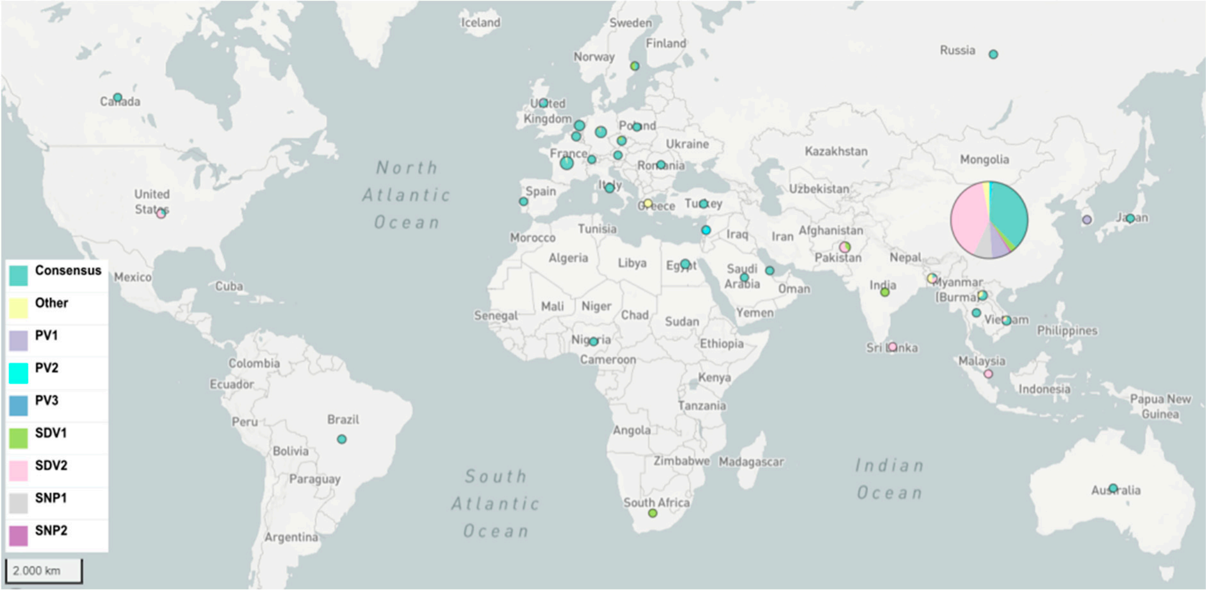

Supplementary Figure S6: Global map showing the frequency of promotor variant in each country for IncHI2 plasmid. The pie chart size increases according to the number of genomes present in the specific country.

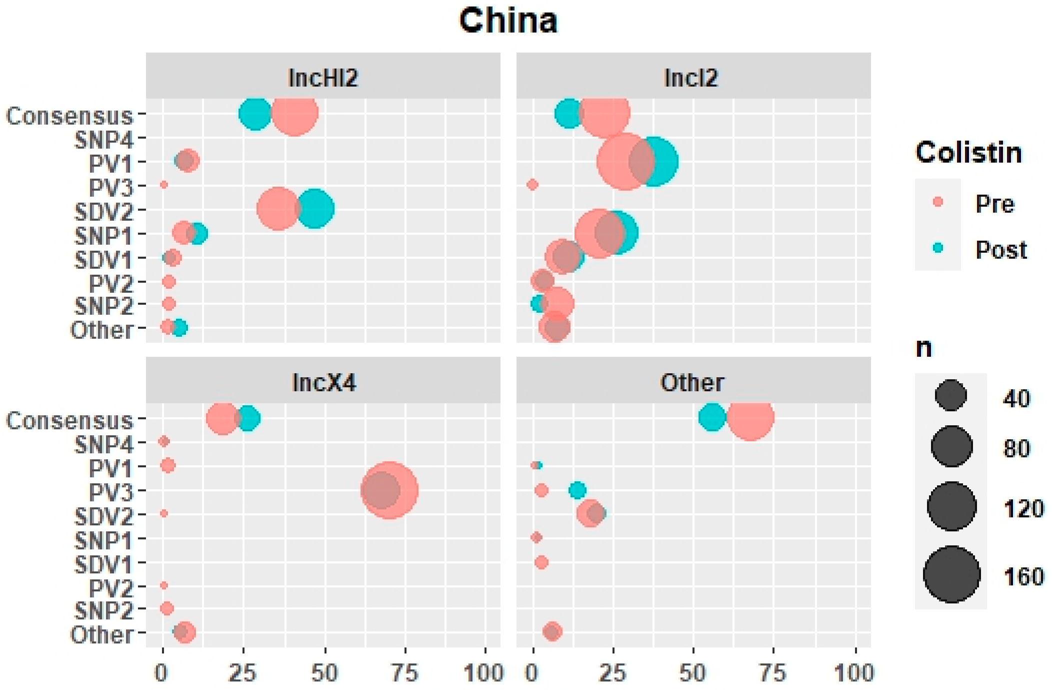

Supplementary Figure S7: Promotor variant frequency comparison between before (red) and after (blue) colistin ban in China split by plasmid type. The size of the circle is directly proportional to the number of isolates of each category.

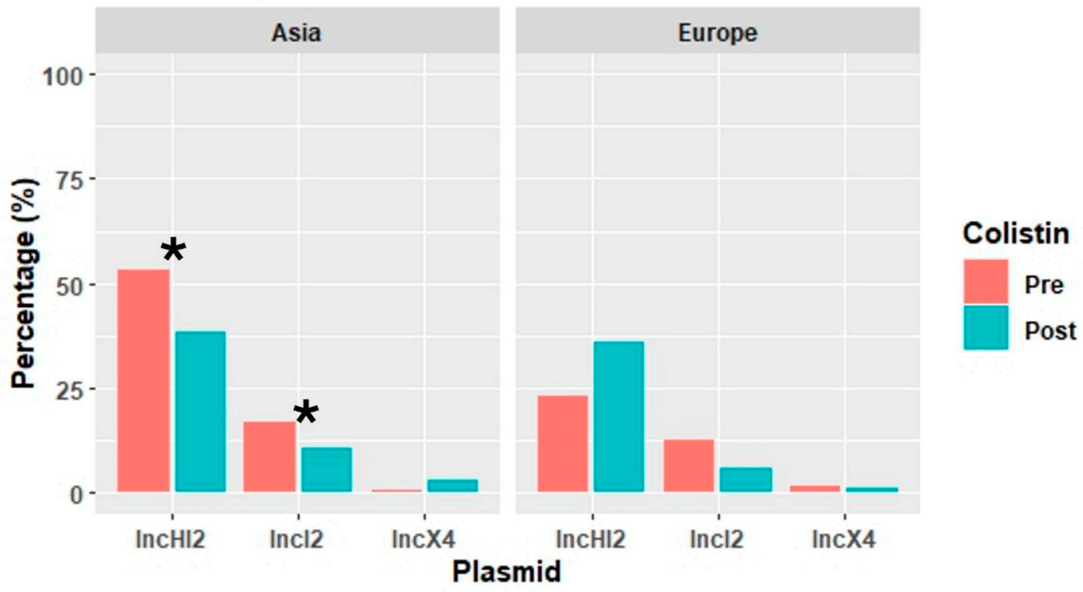

Supplementary Figure S8: ISApl1 presence in Asia and Europe in the distinct plasmids. Bar plot graph showing the percentage of isolates carrying ISApl1 before (red) and after (blue) colistin ban stratified by the three most common plasmids (IncHI2, IncI2 and IncX4). Difference between groups was tested with chi-square test and, when significant differences were found (p-value<0.05) an asterisk (*) was marked.

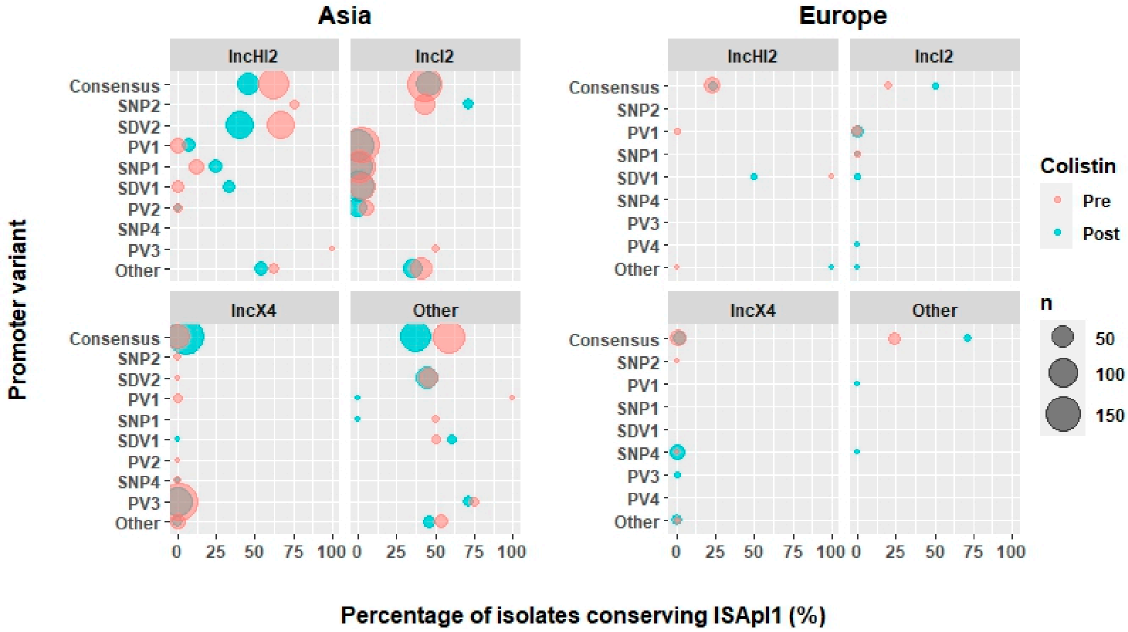

Supplementary Figure S9: Comparison of the percentage of isolates conserving ISApl1 before (red) and after (blue) colistin restriction for each promotor variant in Asia and Europe. The size of the circle is directly proportional to the number of isolates of each category.
